# Fully fluorinated non-carbon compounds NF_3_ and SF_6_ as ideal technosignature gases

**DOI:** 10.1038/s41598-023-39972-z

**Published:** 2023-08-21

**Authors:** Sara Seager, Janusz J. Petkowski, Jingcheng Huang, Zhuchang Zhan, Sai Ravela, William Bains

**Affiliations:** 1 Department of Earth, Atmospheric and Planetary Sciences, Massachusetts Institute of Technology, 77 Massachusetts Avenue, Cambridge, MA 02139 USA; 2 Department of Physics, Massachusetts Institute of Technology, 77 Massachusetts Avenue, Cambridge, MA 02139 USA; 3 Department of Aeronautics and Astronautics, Massachusetts Institute of Technology, 77 Massachusetts Avenue, Cambridge, MA 02139 USA; 4 JJ Scientific, 02-792 Warsaw, Poland; 5 School of Physics and Astronomy, Cardiff University, 4 The Parade, Cardiff, CF24 3AA UK

**Keywords:** Astrobiology, Exoplanets

## Abstract

Waste gas products from technological civilizations may accumulate in an exoplanet atmosphere to detectable levels. We propose nitrogen trifluoride (NF_3_) and sulfur hexafluoride (SF_6_) as ideal technosignature gases. Earth life avoids producing or using any N–F or S–F bond-containing molecules and makes no fully fluorinated molecules with any element. NF_3_ and SF_6_ may be universal technosignatures owing to their special industrial properties, which unlike biosignature gases, are not species-dependent. Other key relevant qualities of NF_3_ and SF_6_ are: their extremely low water solubility, unique spectral features, and long atmospheric lifetimes. NF_3_ has no non-human sources and was absent from Earth’s pre-industrial atmosphere. SF_6_ is released in only tiny amounts from fluorine-containing minerals, and is likely produced in only trivial amounts by volcanic eruptions. We propose a strategy to rule out SF_6_’s abiotic source by simultaneous observations of SiF_4_, which is released by volcanoes in an order of magnitude higher abundance than SF_6_. Other fully fluorinated human-made molecules are of interest, but their chemical and spectral properties are unavailable. We summarize why life on Earth—and perhaps life elsewhere—avoids using F. We caution, however, that we cannot definitively disentangle an alien biochemistry byproduct from a technosignature gas.

## Introduction

The search for signs of life beyond Earth is an increasingly popular scientific research area as telescope capability advances. For exoplanets, the successful launch and operation of the James Webb Space Telescope (JWST)^1^, brings in a new level of precision for exoplanet atmosphere measurements, fostering hope that the community may find signs of life through the detection of exoplanet atmosphere biosignature gases within a decade^2–8^. Yet, as the community pushes deeper into biosignature gas identification, a dawning conclusion is that biosignature gases will always be stymied by false positives, fatal to robust conclusions about the presence or absence of life. Because of this, there is a growing interest in the study of technosignatures as indicators of life beyond Earth.

Technosignatures are astronomically detectable signs of a technological society on an exoplanet^9–14^. The concept of technosignatures dates back decades and includes several categories, such as: Lowell’s canals on Mars^15^; artificial illumination of a planet (e.g.,^16,17^; megastructures (e.g.,^18,19^); waste heat (e.g.,^18,20,21^); and artificial (i.e., non-terrestrial) artifacts (e.g.,^22–24^). Technosignature research has recently been growing, e.g., ^11,12,25,26^.

Technosignature gases, as a subset of technosignatures in general, are artificially produced gases that can accumulate to detectable levels in an exoplanet atmosphere. A technosignature gas can either be emitted for a specific purpose or released as a by-product of industrial civilization^12,16,27^. Proposed technosignature gases center around industrial pollutants such as chlorofluorocarbons (CFCs), hydrofluorocarbons (HFCs), and perfluorochemicals (PFCs)^12,28^. A conclusion from the above references is that detection of CFCs in exoplanet atmospheres, under the most favorable assumptions, would require 100–500 h of JWST in-transit observation time (Table 1). This is increased to 200–1000 h of total observing time, when taking into account an out-of-transit equivalent baseline time. This can be compared to the current JWST time allocation to individual exoplanets which are typically up to 20 h, with a few planet full phase curves allocated around 40–50 h, and one transiting exoplanet atmosphere exception at 70 h^29^. The exception to the long-required observation times is for the hypothetical and highly favorable case of a terrestrial-size planet transiting a tiny star—a white dwarf star which itself is about the size of Earth and is a dead remnant of a Sun-like star. This scenario would require only tens of hours of in-transit time^28,30,31^. See the SI for more details on CFCs and other suggested technosignature gases.

**Table 1:** Summary of proposed exoplanet atmosphere technosignature gases including simulated in-transit observation times.

Technosignature gas	Planet type	Atmosphere type	Star type	JWST detectable abundance	Observation time
CFC 14 (CF_4_)	Rocky-Earth-size	N_2_-dominated	White Dwarf	10 × present Earth’s atm. abundance	40.8 h^28^
CFC-11 (CCl_3_F)	Rocky-Earth-size	N_2_-dominated	White Dwarf	10 × present Earth’s atm. abundance	28.8 h^28^
CFC-11 (CCl3F)	Rocky-Earth-size	N_2_-dominated	M-dwarf	0.225 ppb	100–300 h^12^
CFC-12 (CCl2F2)	Rocky-Earth-size	N_2_-dominated	M-dwarf	0.515 ppb	100–300 h^12^
NO_2_	Rocky-Earth-size	N_2_-dominated	M-dwarf	20 × present Earth’s atm. abundance	*500 h^32^
NH_3_, N_2_O	Rocky-Earth-size	N_2_-dominated	G-dwarf	N/A	N/A^33^

*not explicitly stated whether this is in-transit or both in-transit and out-of-transit time. N/A means no information available.

**Figure 1:**
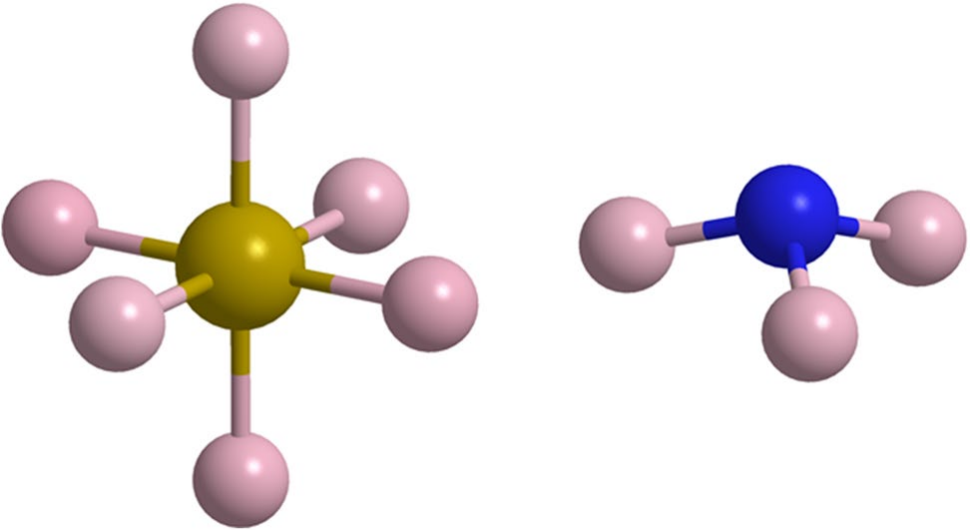
Chemical schematic of the fully fluorinated molecules: SF_6_ (left) and NF_3_ (right). Atoms are depicted by colors as follows. Yellow: sulfur. Blue: nitrogen. Pink: fluorine.

Here we propose nitrogen trifluoride (NF_3_) and sulfur hexafluoride (SF_6_)—fully fluorinated non-carbon compounds—as potential technosignature gases (Fig. 1). A fully fluorinated compound is a molecule where the central atoms are only bonded to fluorine atoms. For example, in NF_3_ the central nitrogen atom can bind to three other atoms. Because each of the bound atoms is fluorine, we call NF_3_ fully fluorinated. NF_3_ and SF_6_ have been only briefly mentioned as technosignatures^34,35^, the case has not previously been developed.

We are motivated to explore non-carbon fully fluorinated compounds primarily because life on Earth never makes compounds containing the N–F and S–F bonds, nor fully fluorinated non-carbon compounds ("Results" section). S–F and N–F bonds are not even made as metabolic intermediates and are likely to be universally excluded by life, no matter its biochemistry. In fact, life on Earth very rarely uses F chemistry. Only a few species make C–F bonds at all (Petkowski et al. in prep.). While life on Earth does not make fully fluorinated carbon molecules (e.g., tetrafluoromethane, CF_4_, or CFCs that have been previously considered as potential technosignature gases^12^), life could in principle do so without inventing completely new enzymes and other necessary biochemical repertoire (Petkowski et al. in prep.). Hence fully fluorinated non-carbon molecules may be more robust technosignature gases than (fully) fluorinated carbon molecules.

**Figure 2:**
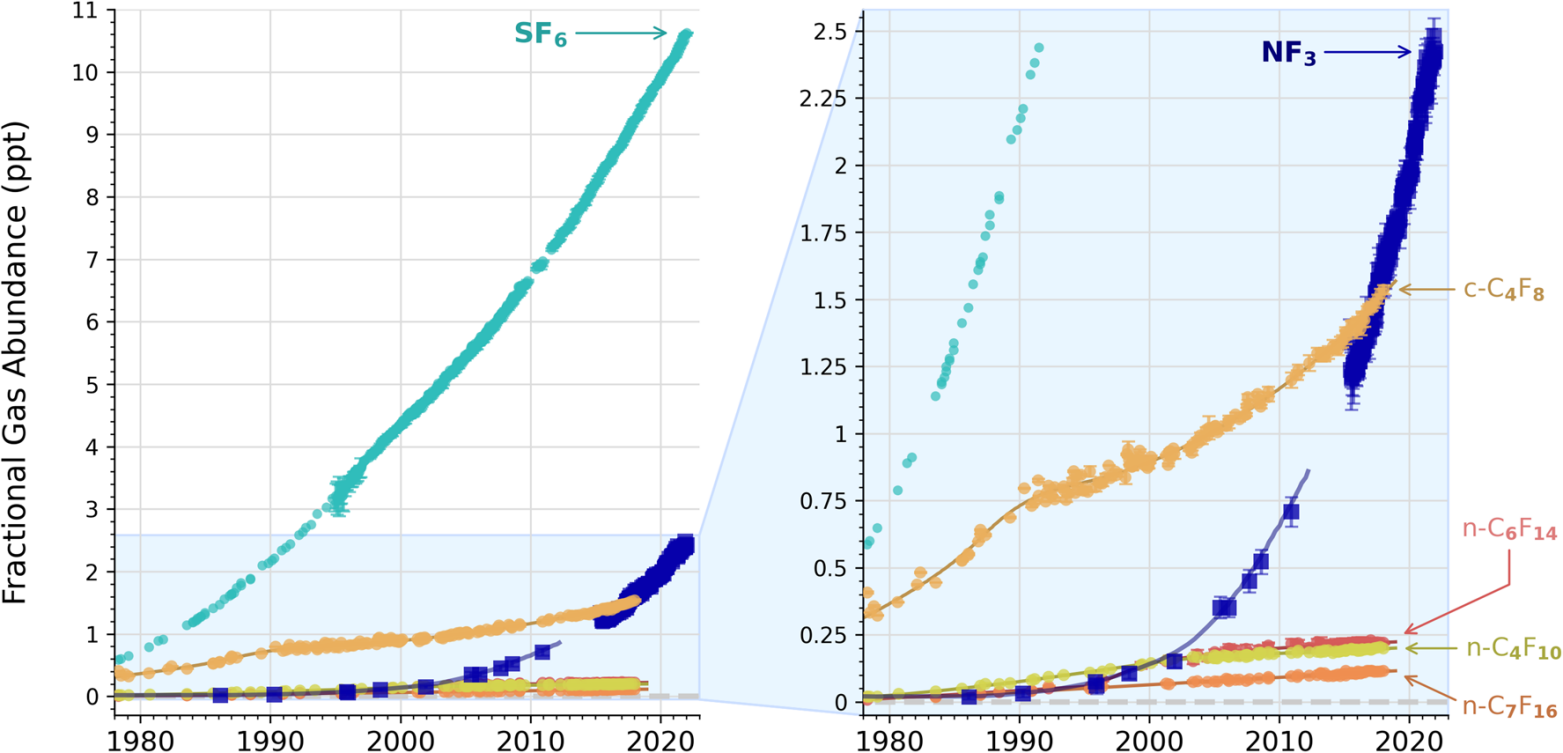
Atmosphere gas abundance for some industrial pollutants including NF_3_ and SF_6_. The y-axis is the fractional gas abundance in part-per-trillion (ppt) by volume and the x axis is time in years. Both NF_3_ and SF_6_ have a rapid increase compared to other industrial pollutants. The right panel is a zoomed in version of the left panel. Data from^36,37,95^, and notably 2013–2022 data is from the Global Monitoring Laboratory (GML) (SF_6_: https://gml.noaa.gov/hats/combined/SF6.html; NF_3_: https://gml.noaa.gov/hats/gases/NF3.html).

SF_6_ and NF_3_ on Earth are not only industrial pollutants but their relative atmospheric abundance has rapidly increased^36,37^ (See Fig. 2 and SI). NF_3_ has no known abiotic sources and was entirely absent from the pre-industrial atmosphere^37^. Within the last half century, the NF_3_ abundance in the atmosphere rose to close to 3 part-per-trillion (ppt) by volume (Fig. 2). SF_6_ does not have significant abiotic sources that could mimic its rapid increase in the atmosphere. Like NF_3_, the steady and rapid relative increase of SF_6_ in the atmosphere from very low background abundances of < 0.06 ppt to around 11 ppt in the last half a century combined with its relatively long atmospheric lifetime of at least a couple of hundred years^38^ further supports SF_6_ as a good technosignature gas candidate. However, we caution that the atmospheric chemistry of NF_3_ and SF_6_ has not been well studied and many potential destruction pathways for NF_3_ and SF_6_ may not be known (see Tables S2 and S3).

## Results

### No fully fluorinated molecules are made by life

Life on Earth is not known to produce any molecules with N–F or S–F bonds, and this includes fully fluorinated N and S compounds. We derived this result from our natural products database which is a curation of all known biochemicals and natural products (i.e. compounds produced by life) from an extensive literature online chemical repository search ("Methods" section and^39,40^). Here, "natural products" means chemicals made by life.

Life does produce some compounds with N–Cl, N–Br, S–Cl, and S–Br bonds, but none are volatile. In addition, the N–Cl, N–Br, S–Cl, and S–Br compounds are typically intermediaries and not molecules that accumulate on their own. The molecules containing N–Br and N–Cl are quite reactive and therefore rare, a notable example being a neurotransmitter N-bromotaurine^41^ and pseudoceratonic acid^42^. The S–Cl and S–Br bond-containing molecules have been found only in proteins as intermediaries in synthesizing the N–S bonds^39^.

**Figure 3:**
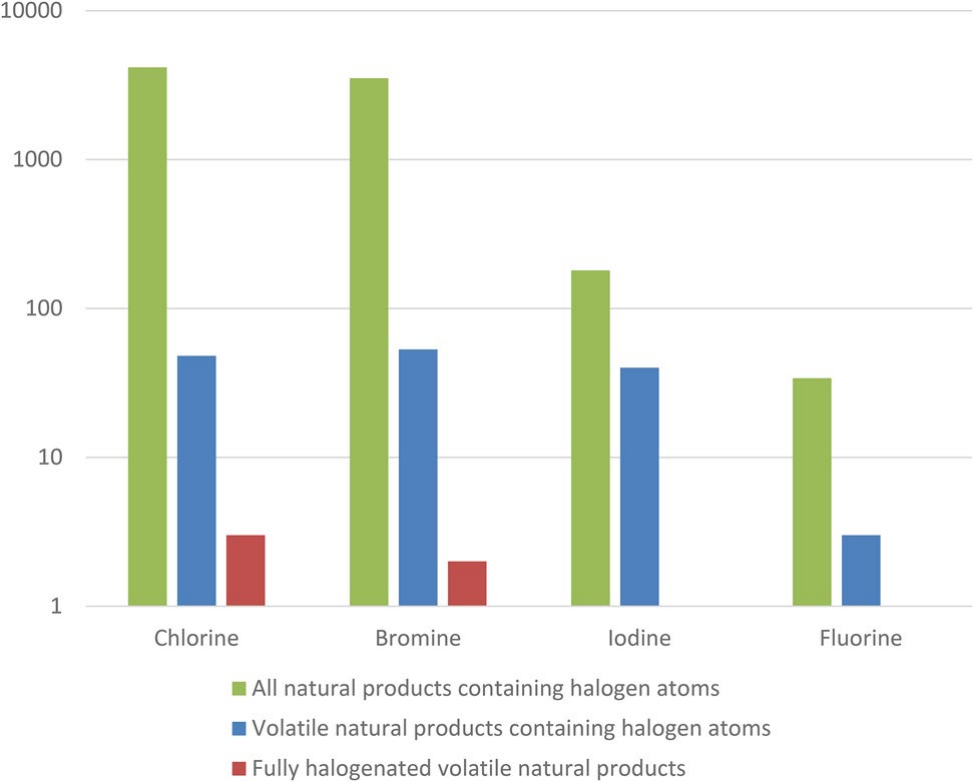
Number of molecules containing C–X bonds produced by life (called “natural products”), where X is Cl, Br, I, or F. For comparison the numbers are separated into three categories. Green: all known natural products in the category (i.e. produced by life). Blue: the subset of volatile natural products, here limited to molecules with 6 non-H atoms or less. Red: the subset of fully halogenated volatile natural products, also limited to molecules with 6 non-H atoms or less. Life rarely produces fully non-F halogenated volatile natural products and does not produce any fully fluorinated products of any kind (C–F, N–F, S–F, or other). Note that one fully halogenated molecule is double counted as it contains both Cl and Br, bromotrichloromethane. Not shown is that life does not produce any molecules with N–F or S–F bonds.

In comparison with the rare N–Cl, N–Br, S–Cl, and S–Br containing molecules, there are thousands of compounds containing C–Cl, C–Br and C–I bonds that are made by life (Fig. 3). Most of the life-produced halogenated compounds are Cl-containing compounds (~ 2% of all known natural products, where the current known total number of unique natural products is ~ 220,000). Br-containing compounds produced by life are a close second (~ 1.7% of all known natural products). Iodine-containing natural products are much more rare but still a significant group with approximately 200 known examples (~ 0.1% of all known natural products). The above includes solids, liquids, and gases; it is worth noting that all of the volatile halogenated (Cl-, Br, I-containing) compounds produced by life are halocarbons, where the halogen atom is directly bonded to carbon.

In contrast to the thousands of Cl-, Br- and I-containing carbon compounds made by life, the F-containing compounds are nearly excluded from life’s repertoire, numbering only 34. And, only two of these 34 are volatile. Nearly all of the known biogenic F-containing natural products are fluorinated carboxylic acids (Petkowski et al. 2023 in prep.). There are no fully fluorinated C–F compounds known to be produced by life.

Although life does not produce fully fluorinated molecules, life does actually produce at least four carbon compounds that are fully halogenated with halogens other than fluorine. This is a very small percentage of all volatile halocarbons produced by life. For some numbers, and considering molecules with 6 or fewer non-hydrogen atoms, there are 85 volatile halocarbons produced by life. There are 34 possible halomethanes, of which 14 are fully halogenated. 22 halomethanes are produced by life on Earth^40^. Out of those 22, only 3 are fully halogenated: tetrachloromethane (CCl_4_) produced by several plants and marine algae^43^, tetrabromomethane, CBr_4_, produced by from various marine algae, e.g. *Asparagopsis taxiformis*^44^*,* and bromotrichloromethane, CBrCl_3_, that contains both Cl and Br atoms, produced by marine algae^44^. For completeness, the fourth fully halogenated carbon compound produced by life is tetrachloroethene (C_2_Cl_4_), produced by Hawaiian red seaweed *Asparagopsis taxiformis*^44^.

Again, the number of fully fluorinated molecules made by life is zero, no matter if they are fluorocarbons or if the F atom is attached to a non-carbon element.

We explain why life avoids F-containing compounds in Petkowski et al. 2023 (in prep.) and briefly summarize the explanation here. We have identified three challenges that F chemistry poses for life on Earth that make the use of fluorine in Earth’s biochemistry a difficult prospect:

1. Relatively low bioavailability of F, which is primarily locked inside insoluble minerals, and is not available in surface water (unlike Cl, Br and I).
2. The uniquely high electronegativity of F, which means that enzymes that handle Cl, Br, peroxide and other oxidizing species cannot be repurposed to handle F. As a result of this challenge life needs to evolve a completely novel enzymatic machinery to create C–F bonds.
3. The lack of reactivity of the C–F bond, which makes evolving catalysts that can handle C–F bonds a difficult task.

These substantial evolutionary barriers mean that almost all life has found ways to address its ecological requirements with chemistry other than C–F chemistry. All three factors are general properties of fluorine chemistry, are not specific to terrestrial biochemistry and therefore are likely universal.

### NF_3_ and SF_6_ have unique spectral features compared to dominant atmospheric gases

**Figure 4:**
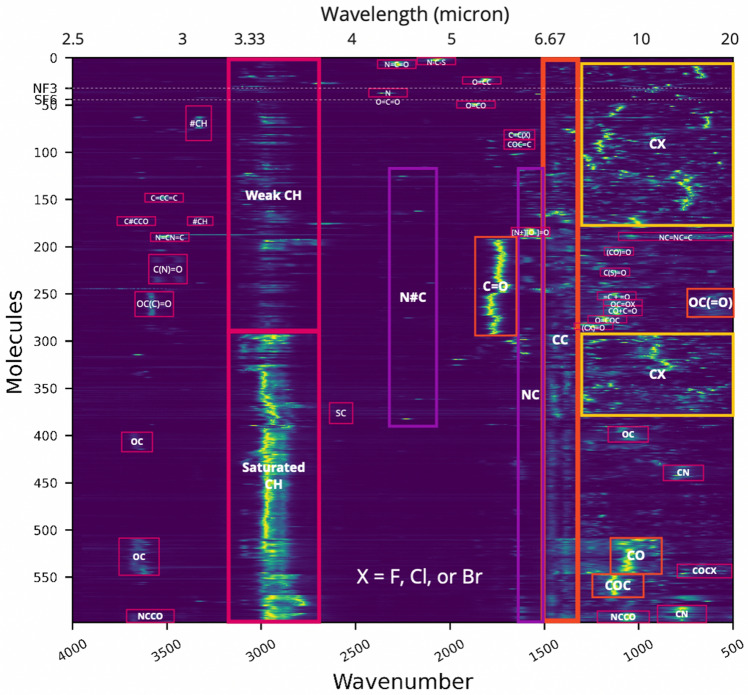
The molecular spectra phalanx plot compares the absorbance spectra amongst molecules. The x axis is wavenumber ranging from 4000 to 500 cm^−1^ (2.5–20 μm). The y axis is the order of the molecules. Color represents the intensity of the absorption peaks; yellow and green represent strong absorption, while blue and purple represent weak or no absorption. Note the absorbance is normalized to 1. The spectra phalanx provides a visualization of which molecules are clustered together in wavelength based on their spectral data. Functional groups are labeled at their clustering points. The grey dashed lines near the top of the plot mark NF_3_ and SF_6_ and show their relative uniqueness in wavenumber space as compared to other halogenated species. The spectra data are from 600 gas species in our All Small Molecules Database (ASM)^40^ (volatile molecules with up to 6 non-H atoms) that have available spectra in NIST^64^. See “Methods” for details on construction of this figure.

**Figure 5:**
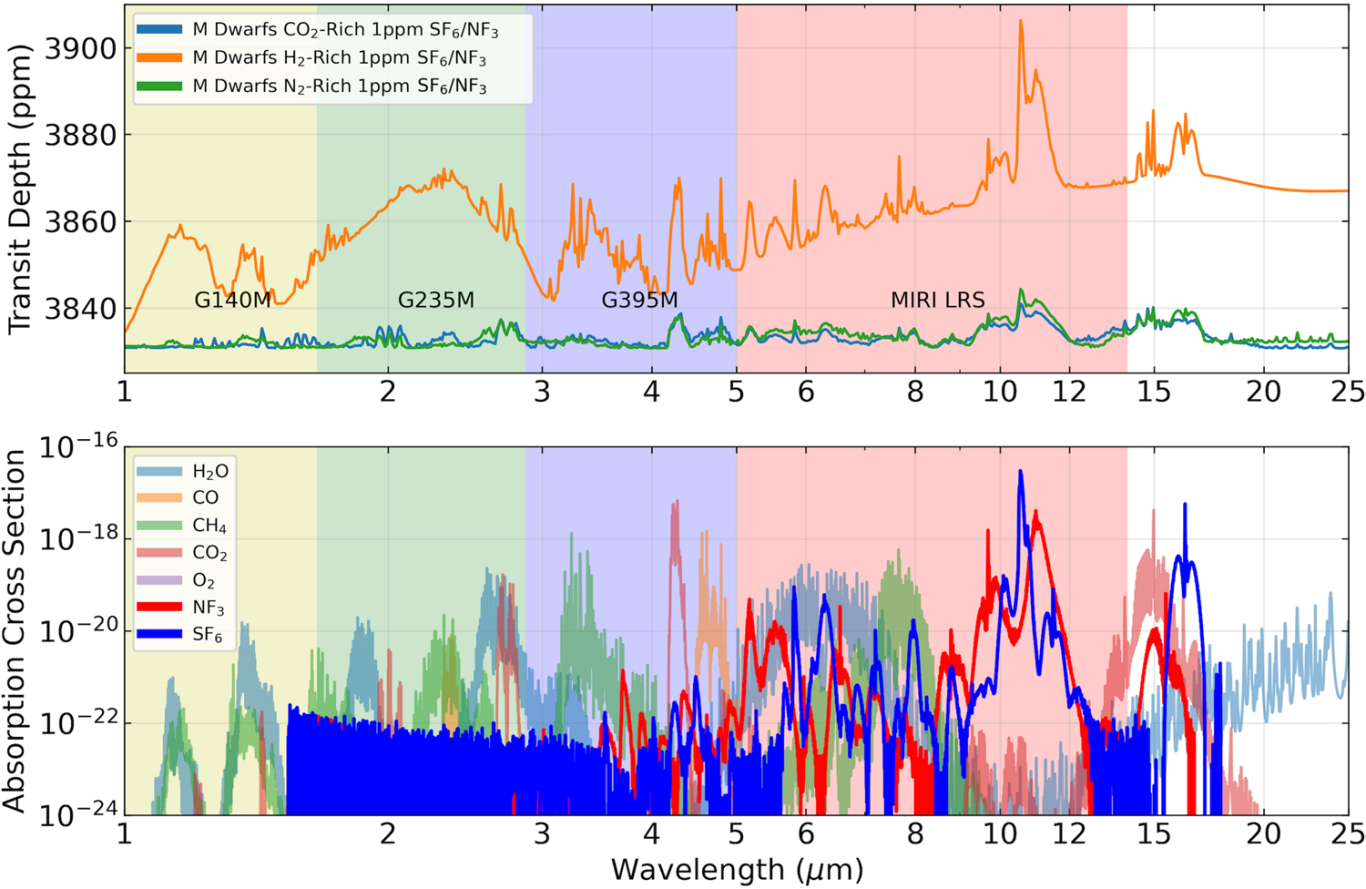
Simulated spectra of an exoplanet with transiting an M5V star with an atmosphere abundance of 1 part-per-million of NF_3_ and SF_6_. Top panel: the y-axis shows transit depth (ppm), and the x-axis shows wavelength (μm). The spectra are simulated from 1 to 23 μm, covering the wavelength span of most of JWST’s observation modes. The yellow, green, and blue regions show the spectral coverage of NIRSpec, and the red region shows coverage of MIRI LRS. Bottom panel: absorption cross sections in cm^2^ as a function of wavelength for key molecules of interest. For context, the contemporary Earth abundance levels are 3 ppt^37^ and 11 ppt^38^ for NF_3_ and SF_6_ respectively.

The gases NF_3_ and SF_6_ have unique spectral features as compared to bulk terrestrial planet atmosphere gases (Figs. 4 and 5). NF_3_ and SF_6_ absorption fall in the 9–12 micron spectral window where the plausible dominant super Earth or Earth-sized planet atmospheric gases CO_2_, CO, CH_4_, and the strong H_2_O vapor spectral features do not appear. Recall the gases N_2_ and H_2_ have no distinctive spectral features at infrared wavelengths; being homonuclear they have no net dipole moment. We note that the NF_3_ and SF_6_ strong absorbing power in a unique part of the spectrum compared to other major atmospheric gases is why they are potent greenhouse gases here on Earth. Trace gases of interest (such as PH_3_, NH_3_, SO_2_, H_2_S) other than the dominant terrestrial planet gases also do not have overlapping spectral features to NF_3_ and SF_6_ (Fig. 4).

There are gases with overlapping spectral features to NF_3_ and SF_6_, and these are primarily halogenated carbon compounds (Fig. 4). The multitude of possible halogenated carbon compound gases (which could be biosignatures or technosignatures) pose a more complicated situation. Disentangling SF_6_ and NF_3_ from the multitude of possible chlorofluorocarbon gases and those gases from each other, depends on spectral resolution. For SF_6_ there are only a few candidates with similar main spectral peaks at the same wavelengths to NF_3_ and SF_6_. More work is needed to ascertain what is needed to distinguish amongst all of the halogenated gases, considering spectral features of other molecules.

### Atmospheric concentrations needed for detection

On the order of 1 part-per-million (ppm) atmospheric abundance by volume of NF_3_ and SF_6_ produces a ~ 30 ppm signal in simulated transmission spectra for a terrestrial planet transiting an M dwarf star (Fig. 5). This is for an H_2_-dominated atmosphere. For a CO_2_- or N_2_-dominated atmosphere, the signal is much lower, owing to the much smaller scale height for high mean molecular weight gases compared to the low mean molecular weight gas H_2_ (Fig. 5). This finding is consistent with other biosignature gas detection simulations, including the point that detecting a ~ 30 ppm signal will almost certainly take tens of transits or more (e.g.,^2,45–48^), a much larger number than the typically few transits currently allocated for exoplanets with the JWST^29^. For details of simulated detectability including JWST noise floor see, for example^4,49,50^.

However, we again emphasize a major point in favor of NF_3_ and SF_6_ spectral distinguishability in an exoplanet atmosphere is that the 9–12 micron region has no expected major atmosphere gases with strong spectral features—although many trace gases, especially halogenated compounds, have features in this window.

**Figure 6:**
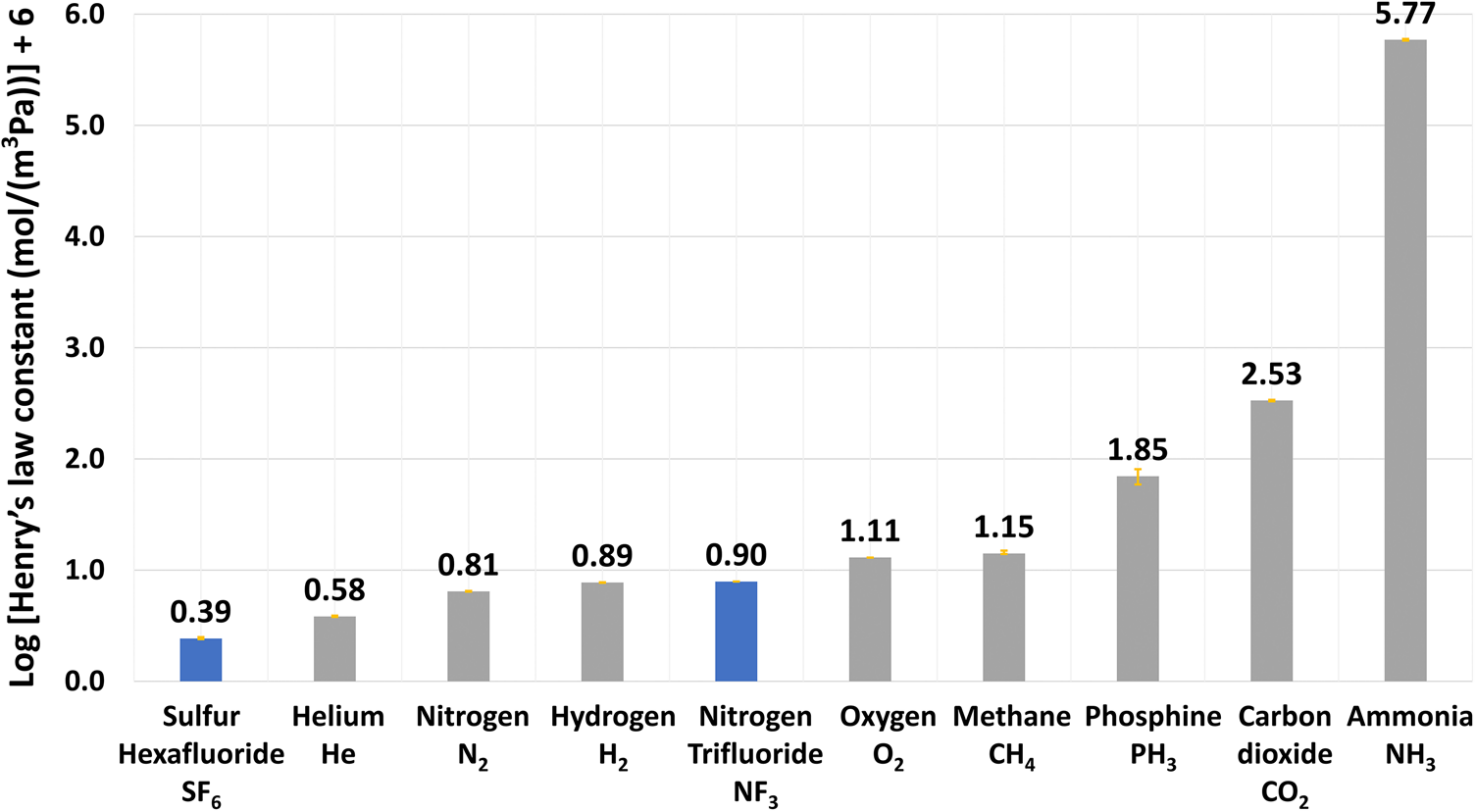
Solubility of various atmospheric gases in water. The x-axis shows the chemical species’ names, and the y-axis shows the Henryʼs law constant on a log scale. Henry’s law is defined as H^CP^_(X)_ = C(X)/p, where H^CP^_(X)_ is Henry’s law constant for a species X in mol Pa^−1^ m^−3^. P is the partial pressure of that species in Pascal, and C(x) is the dissolved concentration (in mol m^−3^) under the equilibrium condition. The larger H^CP^, the more soluble the species is. NF_3_ and SF_6_ have very low water solubility.

The atmospheric accumulation of NF_3_ and SF_6_ is favorable due to their low water-solubility (for a comparison to other gases such as CO_2_ and NH_3_ see Fig. 6). The low water solubility means they will not dissolve in rainwater and fall to the ground or the sea. The NF_3_ and SF_6_ and long atmospheric lifetimes also favor their accumulation (for destruction rates see the SI).

Proper estimation of the lifetime of NF_3_ and SF_6_ would require experimentally measured kinetics of chemical reactions of NF_3_ and SF_6_ in H_2_ at various temperatures. The current thermochemical literature and databases (such as NIST) have very scarce information on reactivity of those species with relevant atmospheric components, at relevant temperatures (Table S1 and S2). Measurement or detailed modeling of those values is essential for progress.

Nonetheless, we can make some estimates. Regarding the lifetime of NF_3_ in an H_2_-dominated atmosphere, the rate constant [cm^3^ molecule^−1^ s^−1^] for the reaction H + NF_3_ → NF_2_ + HF at 300 K has been calculated to be around 2.4 × 10^−20^^51^, which is four orders of magnitude lower than the rate constant of the reaction with the hydroxyl radical (OH), 4.0 × 10^−16^, (reaction OH + NF_3_ → F + H_2_O + NO_2_ in Table S1). This difference suggests that the reaction with OH would dominate over the reaction with an H radical as a main destruction pathway for NF_3_. As a result, the likely lifetime of NF_3_ in an H_2_-dominated atmosphere would not be significantly different than it is in the oxidized, OH-rich, atmosphere of Earth. Both Earth’s atmosphere and the reduced H_2_-dominated atmosphere are expected to be abundant with OH radicals due to photolysis of H_2_O.

Regarding the lifetime of SF_6_, it is a stable and unreactive gas with a lifetime of hundreds to thousands of years in Earth’s oxidizing atmosphere. SF_6_ likely has a similarly long, if not longer lifetime in a H_2_-dominated exoplanet atmosphere. Experimental shock tube dissociation studies of SF_6_ in the presence of H_2_ gas in the temperature range of 1734–1848 K supports this conclusion. The measurements show that H_2_ does not increase the dissociation of SF_6_ as compared to argon gas control^52^. This result indicates that SF_6_ should have at least a similar lifetime in the H_2_-dominated atmosphere as it has in Earth’s atmosphere.

### Abiotic sources and a false positive mitigation strategy

#### NF_3_ has no known abiotic sources

**Figure 7:**
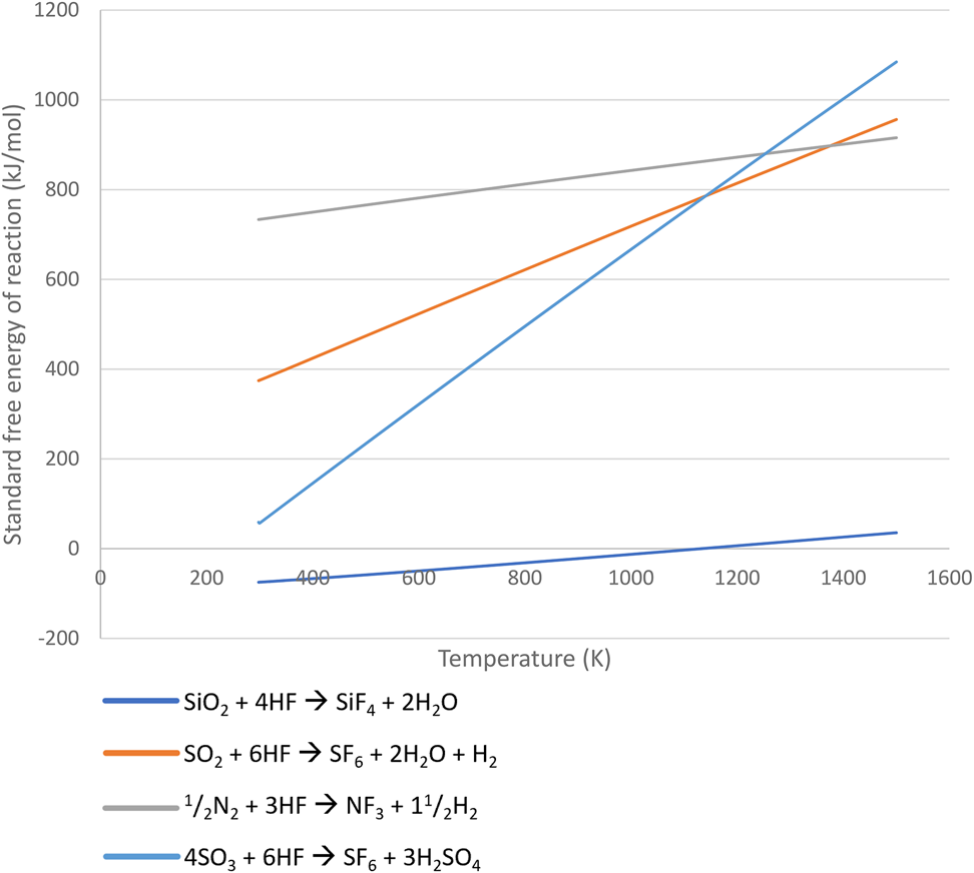
The free energy of formation of NF_3_, SF_6_, and SiF_4_. The y-axis is the standard free energy of formation and the x-axis is temperature (K). The unfavorable thermodynamic conditions (positive ∆G) of the formation of SF_6_ and NF_3_ at all relevant temperatures are in contrast to the favorable abiotic formation of SiF_4_ (negative ∆G), where only SiF_4_ is a known volcanic product on Earth. The formation of NF_3_ and SF_6_ is highly thermodynamically unfavorable and therefore unlikely to be anything but a trace product of planetary geology or volcanism.

NF_3_ has no known abiotic sources. In other words, NF_3_ is not known to be a product of any photochemical, volcanic, or other geological process. We further show formation of NF_3_ is thermodynamically unfavorable for terrestrial planet conditions (Fig. 7). NF_3_ is also not known to be released from any fluorine-containing minerals. The absence of false-positives for NF_3_ is supported by the lack of detection of this gas in any available pre-industrial samples^37^. With no know abiotic or biotic sources, NF_3_ is a, if not the, prime candidate for a technosignature gas search.

#### Abiotic sources of SF_6_

On Earth trace amounts of SF_6_ exists in volcanic rocks in rift zones, faults, igneous intrusions, geo-thermic areas and diagenetic fluids^53^. SF_6_ is predominantly present in fluorites and some granites, while, for example basalts do not contain detectable SF_6_^53,54^.

The exact mechanism of abiotic formation of SF_6_ on Earth is unknown. It is also unclear if SF_6_ is directly made by volcanoes on Earth or its release is just associated with volcanic activity. Harnisch and Eisenhauer have examined the gases from several volcanic fumaroles, e.g., Etna (Sicily), Vulcano Island (Sicily), Kuju (Japan), and Satsuma Iwojima (Japan), and find that they are not significant sources of SF_6_^54^. However, the authors note that the underlying rocks of these volcanoes are not granitic and as a result might lack a source for SF_6_^54^. The equilibrium pre-industrial atmospheric concentration of SF_6_ on Earth is estimated to be < 0.06 ppt^53^. The dominant F-containing volcanic gas on Earth is HF with abundances reaching 0.5–15 ppb^55^. Other trace F-containing species, including NH_4_F, SiF_4_, (NH_4_)2SiF_6_, NaSiF_6_, K_2_SiF_6_, KBF_4_, and organo-fluorides, are also associated with volcanic activity or released by volcanoes, but to a much lower extent than HF^56–59^. It is therefore unlikely that SF_6_ will be a significant false-positive on a water-rich terrestrial planet as Earth.

The hypothesis that volcanic SF_6_ is negligible on Earth is also supported by thermodynamics of SF_6_’s formation (Fig. 7). The formation of SF_6_ (and NF_3_) is highly thermodynamically unfavorable and therefore unlikely to be a source of anything but a trace product of planetary geology or volcanism. The unfavorable thermodynamic conditions of the formation of SF_6_ (and NF_3_) are in contrast to the abiotic formation of SiF_4_, another non-carbon fully fluorinated gas, that is a known volcanic product on Earth^60^ (Fig. 7).

#### Strategies to rule out SF_6_ (and NF_3_) false positives

We first ask if SF_6_ could be a significant abiotic gas on a planet with an environment different from Earth. The answer is yes, as follows. On a dry planet, or a planet that is otherwise H-depleted one would expect a different profile of F-containing volatiles erupted by volcanoes than on Earth which could lead to a potential false-positive interpretation of the source of the detected SF_6_. On a H-depleted planet, fluorine will be bound to a greater extent to elements other than H. Therefore HF, while still expected to be erupted by volcanoes, would not dominate volcanic gases. Such a scenario opens the possibility for SF_6_ to be a much more abundant volcanic product on dry exoplanets than it is on wet planet Earth.

The view that SF_6_ could be a volcanic product on an H-depleted world is supported by the tentative detection of 0.2 ± 0.1 ppm SF_6_ in the atmosphere of Venus by Venera 14^61^. If correct, this value is five orders of magnitude larger than the amount of SF_6_ detected in Earth's atmosphere. Since Venus’ crust and atmosphere are significantly H-depleted (though the deeper mantle may be relatively less H-depleted^62^), it is likely that the majority of F is erupted as other compounds than HF, e.g. SSF_2_, COF_2_, FClCO, and SOF_2_, etc.^63^. Therefore, it is not unexpected that in the H-depleted environment of Venus, with abundant sulfur, SF_6_ could also be a volcanic product released in significantly higher abundance than on Earth. SF_6_ could also be the result of weathering of fluorite minerals which abundance on Venus is poorly constrained.

We now turn back to non-H-depleted planets that are the focus of this paper, and propose a strategy to rule out any volcanic origin of SF_6_. We propose simultaneous observations of SiF_4_ to be employed as a method to rule out volcanic sources of SF_6_ (and NF_3_). The overview reason is that SiF_4_ is much more thermodynamically favored over SF_6_ (see SI Section 4) such that any volcanic activity that produces SF_6_ will produce significantly higher amounts of SiF_4_. In more detail, SiF_4_ is a known volcanic gas on Earth. Volcanic production of SiF_4_ can reach several tons per day and in some instances, such as in the Satsuma-Iwojima volcano plume, SiF_4_ production can rival that of HF^60^. As with SF_6_, the formation of SiF_4_ will be favored in hydrogen-depleted environments and low temperature gas sources. It is conceivable that on a planet with much more active low-temperature volcanism and lower crustal and atmospheric H abundance than Earth that a larger quantity of SiF_4_ would be released by volcanoes into the atmosphere. SiF_4_, therefore, can be an indicator of geological activity on a planet with volcanic chemistry much more favorable to forming SF_6_ than Earth.

In an event where SF_6_ is detected but simultaneous observations of SiF_4_ is not, the likelihood increases that SF_6_ is biological or technological. This conclusion is supported by our calculations that suggest that there are no plausible conditions (pressure 0.1–10,000 bar, H_2_O content 0.1–95%, mantle redox state (MH vs. IW etc.)) where the amount of SF_6_ (or NF_3_) produced by volcanoes comes to within ten orders of magnitude of that of SiF_4_, effectively ruling out the possibility of volcanically-driven co-existence of SiF_4_ and SF_6_ (or NF_3_) (Figure S1 and Figure S2). All of the above rationale applies to NF_3_; unlike SF_6_ there are no abiotic sources on Earth, and so volcanism is even less likely to make this gas on another planet.

There appears to be no spectral information spectral information for SiF_4_^64^, getting such information is crucial for the execution of our proposed mitigation strategy. We conclude this section with a call to study the spectroscopy of the fully fluorinated non-carbon molecules.

## Discussion and summary

### Prospects

The JWST has been operational for science as of 2022 and is our best currently existing capability for exoplanet atmosphere observations for transiting planets via transit transmission spectroscopy. Two categories of terrestrial planet atmospheres are accessible by JWST. The first is the not yet existing terrestrial planets transiting white dwarf stars (See "Introduction" section). The second category is terrestrial planets transiting small red dwarf stars. In addition to the long-required observation times for technosignature gases for such systems ("Introduction" section), a major challenge is the red dwarf host star variability (e.g.^65^ and references therein). Stellar variability is a collective term for a rich set of physical phenomena caused by stellar surface inhomogeneities which come in the form of granulation and magnetic features such as spots and faculae (e.g., ^66^). The host star variability changes with time as active regions evolve and as the star rotates. Stellar spots and faculae have different temperatures from the disk-averaged photosphere, and for cooler stars, have molecular features distinct from the star itself but similar to those in a planet’s atmosphere (^65^ and references therein). Stellar surface inhomogeneities can induce rogue features in stellar data which mimic signatures of exoplanet atmospheres; some studies have found that the signal from stellar inhomogeneities exceeds the signal from the planetary spectral features (e.g.,^65,67,68^). A community of over 100 experts has summarized the challenges and potential mitigation strategies of M dwarf host star variability effects on small transiting exoplanet atmospheres (NASA's Exoplanet Exploration Program Study Analysis Group 21 (SAG21)^65^).

New large ground-based telescopes now under construction are planned to be online within the next decade: the Extremely Large Telescope (EELT, 39 m aperture diameter)^69,70^; the Thirty Meter Telescope (TMT, 30 m aperture diameter)^71^; and the Giant Magellan Telescope (GMT, 20 m aperture diameter)^72,73^. These large telescopes can directly image habitable-zone planets orbiting (i.e., not necessarily transiting) M dwarf stars with the right coronagraph instrumentation and extreme adaptive optics. The challenge is overcoming the high planet-star contrast at 10^7^–10^8^ levels. METIS^74^ on the EELT will have mid-infrared direct imaging capability via a coronagraph and extreme adaptive optics that includes low and medium resolution spectroscopy; however METIS is designed for planet discovery via imaging and its limited sensitivity means METIS is unlikely to enable reliable ~ 10 micron spectrum of a temperate, rocky world orbiting an M dwarf star (^74^ and Quanz, priv. comm. 2023). Direct imaging in the thermal infrared for about five Sun-like stars may also be possible^74,75^. Near infrared imaging and spectroscopy of reflected light from rocky planets in their host star's habitable zone is anticipated to be possible for up to 100 nearby low-mass stars (primarily mid M dwarf stars)^75–77^; while the near-IR is outside of the wavelength range of interest for NF_3_ and SF_6_, such programs may help discover new suitable planets for follow up atmosphere observations. Other than direct imaging, the large ground-based telescopes might be capable of a combination of high-dispersion, high-spectral resolution (R ~ 100,000) spectroscopy with moderate high-contrast imaging to observe spectra of a few rocky planets orbiting Sun-like stars^78^. (Note that not all IR wavelength regions are not easily accessible from Earth’s surface due to Earth’s atmospheric gases).

NASA’s Habitable Worlds Observatory (HWO; https://www.greatobservatories.org/irouv) is a NASA Great Observatory intended to be ready for launch in the mid-2030s and will be designed to directly image exoplanets orbiting Sun-like stars for both discovery and atmospheric characterization. This telescope will have a primary mirror about 6-m in diameter and is planned to have a coronagraph to block out starlight so the planet can be directly imaged. Because HWO will operate at visible to near-IR wavelengths, HWO will not be able to access the infrared windows (> 4 microns) appropriate for NF_3_ and SF_6_ spectral features.

The space-based interferometer under study called the Large Interferometer for Exoplanets (LIFE;^79^) is designed to observe at 4–18.5 microns, and this includes the infrared windows for NF_3_ and SF_6_ spectral features (Fig. 5). LIFE would have four elements each with aperture 2–3.5-m diameter as well as a combiner spacecraft^80^.

### Are SF_6_ and NF_3_ alien biosignatures or universal technosignature gases?

The exclusion of N–F and S–F compounds from Earth biochemistry, the low water solubility of NF_3_ and SF_6_, their unique spectra, long atmospheric life time, and industrial utility on Earth all makes NF_3_ and SF_6_ attractive targets as technosignature gases.

We discuss the reasons for and against NF_3_ and SF_6_ being produced by an alien biology versus by a technological society.

Life on Earth has apparently never made an N–F or an S–F bond-containing molecule, despite life independently evolving F-handling enzymes several times over the last three billion years (Petkowski et al. 2023, in prep.). Life also rarely makes fully halogenated molecules, with only four known examples ("No fully fluorinated molecules are made by life" section). This suggests that there is at least one major evolutionary barrier to making NF_3_ or SF_6_, which would require a correspondingly powerful selective benefit to overcome.

SF_6_ is a completely chemically inert gas, making it essentially “invisible” to biochemistry. Therefore, we have to ask why life, no matter its biochemical makeup, would invest significant metabolic machinery and energy into making a compound that it then throws away, and which, due to its chemical inertness, has a negligible effect on the organism’s immediate environment or on its competitors. Biological production of SF_6_ would cost an organism a large amount of energy while not giving any evolutionary advantage (i.e., does not provide any obvious fitness gain).

NF_3_ is mildly reactive, and so we may speculate that NF_3_ could have the same ecological role as CH_3_Br on Earth, as a mildly reactive, non-specific, diffusible toxin to intoxicate the competition in the immediate environment. Therefore, NF_3_ production could increase the fitness of the NF_3_-producing organism. The toxicity of NF_3_ can be to an extent similar to the toxicity of phosphine (PH_3_)^81^. PH_3_ does not react all that much with biological tissues, but it is toxic to humans, and many other O_2_-dependent organisms, because it converts hemoglobin to methemoglobin (which cannot bind oxygen), i.e. PH_3_ is toxic to (large, terrestrial) oxygen-dependent organisms but not to anaerobic ones^82^. Oddly, in identifying a technosignature gas, a biological source may be a false positive. Against this, we note that there are many mildly reactive, toxic carbon-containing compounds that can be made with the enzymatic machinery that any carbon-based life is likely to possess, such as CH_3_Br, CH_3_I, cyanogen, CO, formaldehyde, nitric oxide, so NF_3_ would not provide any unique advantage over these more evolutionarily accessible substances.

One could argue that the biological repertoire of gases on other planets may surprise us because we have no idea what gases non-Earth-like life might produce^40^. Production of certain biochemicals is often an evolutionary accident or depends on the planetary environmental history. An example is the gas stibine, SbH_3_, which would not be expected to be made by Earth life because Sb itself is a rare element in the Earth’s crust, but nevertheless is synthesized by terrestrial anaerobic sewage sludge microflora^83^. Another example is trimethylbismuth (C_3_H_9_Bi) produced by a variety of bacteria in anaerobic conditions (reviewed in^84^). But we favor the point that while it is possible that an alien biochemistry would find use for NF_3_ and SF_6_, virtually any combination of physical and chemical properties of NF_3_ or SF_6_ can be duplicated with less energy and less dangerous radicals using the chemistry of other halogens and carbon. Indeed, our premise of promoting NF_3_ and SF_6_ as technosignatures is that F is nearly excluded by life on Earth (Petkowski et al. 2023, in prep.)) and that such an exclusion may well be universal.

We further argue that industrial use of some chemicals may be likely to be universal. SF_6_ in particular has unique properties that make it useful for a technological civilization (reviewed "Results" section) and specifically its high dielectric constant and high breakdown voltage as a gas make it valuable in high voltage electrical equipment^36^. Biosignature gases are the product of evolutionary contingency and are limited by thermodynamics and the reactivity of materials to water. Industrial chemicals, however, are the result of an informed, systematic search of all possible chemicals for materials that have optimal properties for a specific application, largely regardless of the thermodynamics of their synthesis or whether their synthesis requires chemistry that is incompatible with earth surface conditions (for example, industrial production of NF_3_ is done in molten ammonium fluoride^85^, an obscure material unlikely to occur on its own on any rocky planet). It is therefore plausible to suggest that an extraterrestrial civilization that wanted, for example, a gaseous product to act as a high voltage insulator and arc quencher, would choose SF_6_, no matter what the entity's own biochemistry, evolutionary history or planetary environment was.

If we in the future have a way to detect the tiny part-per-trillion amounts elsewhere that we humans have accumulated in our atmosphere, and are lucky to catch the small window where a society becomes industrial, we may observe the rapid, steady increase of the atmospheric abundance of SF_6_ or NF_3_. A rapid, steady increase would favor a technological source and may be a solid discriminator between bio- and a true technosignature gas. Even the relatively rapid increase in atmospheric O_2_ that led to the Great Oxygenation Event still took 1–10 million years for O_2_ to accumulate in high enough concentrations to have a weathering effect on rocks (e.g.,^86^). A multi-generational observational campaign can further distinguish between SF_6_ and/or NF_3_ as technosignature and not biosignature gases. Observation of a planet over the time span of 2–4 generations (~ 100 years) to monitor the increase of the SF_6_ and NF_3_ gases could strengthen attribution to a technological source, but one would have to get lucky with timing.

We now turn to the requirement that the atmospheric abundance of NF_3_ and SF_6_ needed for detection with the JWST is of the order of 1 ppm, far higher than current atmospheric levels. Here on Earth, no uniquely technological gas has been produced to accumulate to such relatively high amounts in the atmosphere. 1 ppm SF_6_ is 100 times the current terrestrial level, and it would take ~ 700 years of the current emission rate to build that concentration in Earth's atmosphere, taking the SF_6_ atmospheric lifetime of 3200 years. The greenhouse gas effect of such concentrations on Earth would be catastrophic, which suggests that any alien technological society would curtail their emissions before they reached 1 ppm, unless their goal was substantial global greenhouse heating. Indeed SF_6_ has been considered as a terraforming agent on Mars, albeit for humans at our stage of development a prohibitively expensive one^87^.

Fully fluorinated non-carbon containing gases other than NF_3_ and SF_6_ have little known about them (see discussion in the SI; Table S3).

### Summary

In summary, NF_3_ and SF_6_ are appealing technosignature gases primarily because use of F is nearly excluded by the chemistry of life on Earth for fundamental reasons (Petkowski et al. 2023, in prep.), and moreover because molecules with N–F and S–F bonds are not made by life on Earth at all. We have argued that while an alien biochemistry might find use for NF_3_ and SF_6_, such use would have to provide a significant evolutionary gain that offsets e.g. the large energy expenditure for the synthesis and breakage of fluorine-containing bonds. In contrast, industrial use may be universal due to unique physical and chemical properties of NF_3_ and SF_6_ gases (see SI). Therefore, NF_3_ and SF_6_ may be likely to be used by alien industry no matter the biochemical makeup of the alien biology or the particular environmental conditions of the alien planet. We note however that it is likely that the technological stage of the civilization, or time-span at which NF_3_ and SF_6_ are produced in sufficient amounts to be observed is short.

NF_3_ has no known sources other than industrial, while SF_6_ is produced abiotically in extremely small, trace amounts. Their source can be distinguished from e.g. potential volcanic release into the atmosphere by comparison with the known volcanic gas SiF_4_. The lack of known false-positives for NF_3_ and very low abundance of abiotic sources of SF_6_ on Earth supports their potential as a technosignature gases on exoplanets. NF_3_ and SF_6_’s long atmospheric lifetimes and unique spectral features aid their potential detection.

## Methods

### Custom natural products database

To understand the uniqueness of fully fluorinated compounds out of all chemicals made by life (called “natural products”), we use our database of natural product chemicals curated over the last decade. Our database is presented in^40^ and expanded and completed as described in^39^. We created and curated our database by an extensive literature search and by searching available online natural product repositories^39^.

Our natural products database has been rigorously screened to contain only compounds that are a result of natural biochemical processes of a living organism. It also contains biological sources identified for every molecule (i.e., a list of species from which the natural product was isolated).

We emphasize how challenging it is to compile a complete list of all that is known about each natural product. First, no individual database covers more than 20% of the known natural products. Second, because most natural product databases focus on drug design, they include synthetic derivatives of natural products or drug metabolites that often mimic natural molecules while not being true natural compounds themselves. Other databases often include artificial compounds that emerge as a result of “feeding” an organism with a precursor molecule, or completely artificial compounds that have accumulated as contaminants in plants and animals. We manually excluded such compounds from our database. Third, most data sources needed extensive checking and modification due to a range of format differences and coding errors. All of the above problems motivated us to curate our own natural products database (see^39^ for a full description).

### Spectra visualization tool

A key question for any atmospheric trace gas is whether or not its spectral features are distinguishable from features found in other expected atmospheric molecular gases. We introduce a new tool for analysis, detailed in^88^ and summarized here.

We call our new tool a *spectra phalanx plot* (Fig. 4). This plot enables a visual comparison amongst the absorption peaks of each individual molecule’s spectral features, with molecules that share similar spectral features grouped closer together. Each molecule occupies a horizontal line parallel to the x-axis, where the molecule’s spectral features are plotted as a function of wavelength location. We use color to represent the intensity of the absorption peaks; yellow and green represent strong peaks, while blue and purple represent weak or no absorption. Note the absorbance is normalized to 1, so only the ratio between the peaks and the strongest peak is important, and comparing the absolute intensity between molecules is not possible. For this tool, we use the ~ 600 transmission/absorbance data spectra available from NIST^64^ (with a small subset from HITRAN^89^). The order of the molecules is not meaningful other than that molecules with similar wavelengths of spectral features are grouped together.

We generate the molecule ordering in the spectra phalanx plot using hierarchical clustering, a tree-based approach that builds clusters by iteratively grouping two of the closest cluster/elements into the same cluster and organized in a binary tree structure called a dendrogram^90^. We apply hierarchical clustering on molecular spectra to cluster the molecules and validate that the molecules in the same clusters share similar chemical structures using a molecular maximum common substructure search using methods described in^88^.

In more detail, comparing the molecular IR spectra of two molecules expresses how similar the two are. We use the squared Euclidean distance function to compare normalized molecular spectral signatures, each normalized to have unit peak absorbance. An agglomerative clustering process organizes the pairwise symmetric distance matrix between all pairs of molecules into a cluster hierarchy. Using the hierarchy, one can visualize and identify the molecular spectral features that contribute to the relative detectability of molecules. It is these spectral features that one would want to detect. To locate the distinguishing features, we enumerate the molecules row-by-row in the order of their cluster similarities, imaging a molecule’s spectral signature. The one-dimensional image along the x-axis indicates a bright (yellow) color for high absorbance and dark (blue) for low absorbance (in contrast to a curve plot). Most interesting is the pattern that emerges when one looks at all cluster-enumerated molecular spectral signatures. This phalanx plot shown in Fig. 4 shows that normalized peaks (with some spectral width) at specific wavelengths are shared by many molecules, and these do not contribute significantly to the distinguishability and, thus, relative detectability of the molecule. But it also appears, as shown, that specific clusters share peak distributions that are relatively muted or absent in all the others. These are the distinguishing IR signature features that help detect the molecules in the group. To the degree that the number of molecules in a group is small and the spectral features shared by them are exclusive relative to the others in our All Small Molecule (ASM) database^40^, they are highly detectable. See^88^ for more details.

### Atmospheric spectra simulator

We simulate model atmospheres to assess the approximate atmospheric abundance of gases needed for detection with simulated James Webb Space Telescope (JWST) observations. We use the computer model “Simulated Exoplanet Atmosphere Spectra” (SEAS) code from^88^.

We use the molecular mixing ratio profiles and calculate the optical depth of each layer of the atmosphere^88,91^. We calculate the stellar intensity absorption along each path through the planet atmosphere by *A* = *n*_i,j_ *σ*_i,j_ *l*_*i*_, where *A* is absorption, *n* is number density, *σ* is the absorption cross-section and *l* is pathlength. The subscript *i* denotes each layer that the stellar radiation beam penetrates, and *j* denotes each molecule. For the height of each atmospheric layer we adopt scale height of the atmosphere. We calculate the transmittance, *T*, of each beam using the Beer–Lambert Law. Then, we compute the total effective height *h* of the atmosphere by multiplying the absorption *A* = 1 − *T* by the atmosphere's scale height. To connect to observations, we calculate the total attenuated flux as transit depth (*R*_planet_ + *h*)^2^/R_star_^2^ in units of ppm.

We simulate transmission spectra for a hypothetical 1.5 *R*_Earth_, 5 *M*_Earth_ super-Earth transiting an M dwarf-star similar to GJ 876. We choose 1.5 *R*_Earth_ as it is consistent with a rocky planet^92^. We simulate three planetary atmospheres: ones dominated by H_2_, N_2_ and CO_2_. We choose a relatively massive super Earth planet because a more massive planet is more likely to retain an H_2_-dominated atmosphere than a lower mass planet. We choose to simulate a rocky exoplanet with an H_2_-dominated atmosphere because such an atmosphere is favorable for detection by transmission spectroscopy than a higher mean molecular weight atmosphere such as one dominated by N_2_ or CO_2_. We also simulate an N_2_ and a CO_2_-dominated atmosphere. We take the temperature, pressure, and vertical gas abundance profiles from^48^, where the molecular gas abundances were computed from a photochemical equilibrium model^93,94^. We assume varying atmospheric abundances of SF_6_ and NF_3_, from 1 ppb to 1 ppm.

### Supplementary Information

Supplementary Information.

## Data Availability

The datasets used and/or analyzed during the current study are available from the corresponding author on reasonable request.
