# Supplementary material for: Fully fluorinated non-carbon compounds NF3 and SF6 as ideal technosignature gases
Source: Sci Rep. 2023 Aug 21;13:13576. doi: 10.1038/s41598-023-39972-z (PMC10442443; doi:10.1038/s41598-023-39972-z)
Supplement: Supplementary file 1 — Supplementary Information. [file 41598_2023_39972_MOESM1_ESM.docx]

Supplementary Information for

Fully Fluorinated Non-Carbon Compounds NF_3_ and SF_6_ as Ideal Technosignature Gases

Sara Seager^1,2,3^, Janusz J. Petkowski^1,4^, Jingcheng Huang^1^, Zhuchang Zhan^1^, Sai Ravela^1^, William Bains^1,5^

**Affiliations**

^1^ Department of Earth, Atmospheric and Planetary Sciences, Massachusetts Institute of Technology, 77 Massachusetts Avenue, Cambridge, MA 02139, USA

^2^ Department of Physics, Massachusetts Institute of Technology, 77 Massachusetts Avenue, Cambridge, MA 02139, USA

^3^ Department of Aeronautics and Astronautics, Massachusetts Institute of Technology, 77 Massachusetts Avenue, Cambridge, MA 02139, USA

^4^ JJ Scientific, 02-792 Warsaw, Poland

^5^ School of Physics and Astronomy, Cardiff University, 4 The Parade, Cardiff CF24 3AA, UK

*Correspondence: seager@mit.edu

**SI 1 A Summary of Proposed Technosignature Gases**

Multiple studies have examined the detectability of CFCs in exoplanet atmospheres (e.g.,^1,2^). For instance, ^2^ investigated the detectability of CFC-11 (CCl_3_F) and CFC-12 (CCl_2_F_2_) on TRAPPIST-1e. Assuming a James Webb Space Telescope (JWST) Mid-Infrared Instrument low-resolution spectrometer (MIRI-LRS) noise floor of 10 ppm, they found that present-day Earth abundances of these two CFCs could be detected on TRAPPIST-1e, with about 100 hours of JWST observation time^2^. However, assuming a conservative JWST noise level of 50 ppm, they concluded that even CFCs five times the present-day Earth level would not be detectable by JWST, regardless of the observation time ^2^. In another study, researchers investigated the detectability of CFC-11 (CCl_3_F) on an Earth-sized planet transiting a white dwarf star similar to Beta Persei (commonly known as Algol)^1^. They found that CFC-11 at an abundance ten times the present Earth level could be detected by JWST with about 1.2 days of observation time^1^. While no Earth-sized planets transiting bright white dwarf stars have yet been detected, they remain promising candidates for atmosphere study if they exist and can be discovered.

Other proposed technosignature gases include the simultaneous detection of NH_3_ and N_2_O in an atmosphere that also contains H_2_O, O_2_, and CO_2_ as a signature of extraterrestrial agriculture^3^. The gas NO_2_ as an atmospheric technosignature has been proposed as a sign of an industrial revolution, specifically combustion engines^4^. These technosignature gases, however, are far from unique as both the planet and life also produces them. Terrestrial agriculture is an exploitation of the biochemistry of Earth, and so its reliance of exogenous sources of NH_3_ (as fertilizer) and its production of N_2_O might be specific to terrestrial biology, and not a general sign of agriculture. NO_2_ from internal combustion engines assumes a very specific technological trajectory for the planet: the widespread use of a specific type of fossil-fuel-powered transport infrastructure rather than, for example, steam- or electric-powered transport.

**SI 2 An Overview of NF_3_ and SF_6_**

Both NF_3_ and SF_6_ are present in Earth’s atmosphere not from intentional release but from leakage from industrial use. Here we summarize the origin and properties of NF_3_ and SF_6_.

**2.1 NF_3_ and SF_6_ Physical and Chemical Properties**

At room temperature, NF_3_ is a colorless and non-flammable gas. At room temperature, NF_3_ is only slightly soluble in water^5^ and does not react with water or dilute acids^6–8^. NF_3_ is thermodynamically and chemically stable; for example it does not react with most metals below 250 °C^6,9^, but it can act as a potent yet slow oxidizer^8^.

In recent years, NF_3_ has been widely used in the microelectronics and semiconductor industries where it is used as an etchant in producing thin-film-transistor liquid-crystal displays (TFT-LCD), semiconductors, and solar photovoltaic panels (e.g.,^6,9–12^).

Sulfur hexafluoride (SF_6_) is a colorless, non-toxic, and non-flammable gas^7^. At room temperature, SF_6_ is almost insoluble in water; the solubility of SF_6_ is even lower than that of helium (He), one of the least water-soluble gases^5^ (Figure 6 in the main text). SF_6_ is completely chemically inert and non-toxic. At room temperature, it is unreactive towards most metals. It does not react with magnesium (Mg) and copper (Cu) even if they are heated to “red hot” temperatures. It does not react with phosphorus (P) or arsenic (As), nor with hydrochloric acid (HCl), sodium hydroxide (NaOH), or potassium hydroxide (KOH). SF_6_ does not decompose even when heated to 500 °C. In addition, SF_6_ does not react with liquid water or high-pressure steam. SF_6_ will only react with boiling sodium (Na)^7,13–18^. SF_6_’s only toxic effect on mammals, including humans, is when it is breathed in at greater than 80% concentration and so replaces oxygen in inspired air^19^.

The main reason behind the  chemical stability and unreactivity of SF_6_ is a kinetic barrier to its hydrolysis^7^.  SF_6_ has a unique molecular structure among sulfur-containing compounds. SF_6_ is an octahedrally-shaped molecule with six fluorine atoms symmetrically attached to the central sulfur atom (Figure 1 in the main text). As a result, the S atom is sterically shielded by the surrounding F atoms, making it inaccessible to other reacting molecules, e.g., water.

SF_6_ is used in a wide variety of industries, including the electrical power industry, semiconductor manufacturing, and the production of aluminum and magnesium, to name a few^7,20–22^. In the electrical utility industry, SF_6_ is often used as an insulating gas in electrical transmission and distribution equipment, such as current/voltage transformers, circuit breakers, switchgear, and capacitors (e.g.,^7,20,23^). SF_6_’s chemical stability, non-flammability, and non-toxicity add to its usefulness as an excellent insulating gas^7,24^.

As a result of the low reactivity of NF_3_ and the chemical inertness of SF_6_, once released to the atmosphere these gases have very long residence times.

**2.2 A Steady and Rapid Increase of Atmospheric Concentration of SF_6_ and NF_3_ in an Industrialized World**

The widespread use of NF_3_ and SF_6_ due to their unique advantages and pivotal roles across multiple industries has nonetheless raised concerns about their environmental impact. Both NF_3_ and SF_6_ are very potent greenhouse gases (e.g.,^25–27^). NF_3_ has a 100-year Global Warming Potential (GWP) of about 16100, while SF_6_ has a GWP of about 23500^28^. NF_3_ and SF_6_ are more than five orders of magnitude more efficient greenhouse gases than CO_2_. Since NF_3_ and SF_6_ are very stable, they are difficult to break down and remove from the atmosphere, and hence NF_3_ and SF_6_ have very long atmospheric lifetimes. The atmospheric lifetime of NF_3_ is about 500 years, and that of SF_6_ is 850 - 3200 years^28,29^.

Since the use of NF_3_ in specialized industry began in the 1970s, and was later used in more wide-spread applications in the electronic industry in the late 1990’s, the amount of atmospheric NF_3_ has been rapidly increasing and has doubled every five years since the late 20th century (<https://gml.noaa.gov/hats/gases/NF3.html>). In contrast to SF_6_, NF_3_ does not have any known non-human-made source and measurements of air entrapped in ancient ice Antarctica as well as measurements of NF_3_ in archived air tanks filled before 1975, i.e., before the introduction of NF_3_ to human industry, have resulted in undetectable levels of NF_3_. The undetectable levels of NF_3_ in pre-industrial samples suggest that background NF_3_ levels were essentially zero or no greater than the limit of detection of 0.008 ppt^30^. The rapid rise in the global atmospheric abundance and projected future demands of human industry place NF_3_ as the fastest growing contributor to radiative forcing of all the synthetic greenhouse gases by the mid-XXI century^31^. Lack of detectable pre-industrial NF_3_ supports its potential as a technosignature gas and strongly suggests that NF_3_ does not have any significant abiotic source.

The increase in atmospheric SF_6_ follows the same trend as NF_3_. Over the last 70 years, since its first application in industry in 1953, SF_6_’s concentration in the troposphere increased dramatically from approx. 0.05 ppt to > 4 ppt (Figure 2 in the main text)^32^.

There is limited atmospheric chemistry reactivity data for NF_3_ and SF_6_ with potential destruction pathways possibly unknown. The known rates show very low reaction rates with the dominant atmospheric radical OH, and the only significant rates are for gases of low atmosphere abundance (Tables S1 and S2).

**SI 3 Other Fully Fluorinated Non-Carbon Molecules?**

Given how compelling NF_3_ and SF_6_ are as technosignature gases, one should ask if other volatile fully fluorinated molecules are equally promising as technosignature gases.

Other fully fluorinated human-made gases do exist and are worth further exploration as technosignature gases because they do not exist in nature. However, there are a limited number of possibilities, and the ones that have been synthesized have limited data (e.g., reactivity, water solubility, or gas phase spectral feature information). The list includes PF_3_, P_2_F_4_, SeF_6_, or S_2_F_10_ (Table 1)^33^. Out of the list of possibilities (Table S3), SiF_4_ is made by Earth’s volcanoes and therefore not a suitable technosignature gas (see 5.3) and PF_3_ slowly hydrolyzes in water. Despite its reactivity to water, PF_3_ is worth further exploration, but so far, perhaps because it is not useful for industry, there is almost no spectral or any other relevant information on its potential as a technosignature gas. The other gas candidates listed in Table S3 are all highly reactive in water and not worth further comment, except to note that for some gases life does produce the hydrogenated versions, such as NH_3_, H_2_S, PH_3_, GeH_4_, but never partially fluorinated versions.

| **Reactants** | **Products** | **Rate Law [cm^3^/molecule s]** | **Valid Temperature  Range [K]** | **Rate at 298K** | **Source** | **Note** |
| --- | --- | --- | --- | --- | --- | --- |
| NF_3_ + N | NF_2_ + NF | 2.13x10-12 (T/298)^1.97^ exp(-15154.3/T) | 200 - 4000 | 1.98x10^-34^ | NIST |  |
| NF_3_ | NF_2_ + F | 6.81x10^-8^ exp(-24174.7/T) | 1100 - 1800 | N/A | NIST | High temp rxn |
| OH + NF_3_ | F + H_2_O + NO_2_ | <4.0x10^-16^ | 298 | 4.0×10^-16^ | NIST | Products unknown |
| CF_3_ + NF_3_ | CF_3_NF_2_ + F | 1.99x10^-14^ exp(-3740.5/T) | 303 - 423 | N/A | NIST |  |
| CH_3_ + NF_3_ | CH_3_F_2_N + F | 7.09x10^-14^ exp(-4899.9/T) | 374 - 467 | N/A | NIST |  |
| C_2_H_5_ + NF_3_ | C_2_H_5_F_2_N + F | 2.41x10^-11^ exp(-8300/T) | 410 - 486 | N/A | NIST |  |
| O(1D) + NF_3_ | NF_2_ + OF | 1.1x10^-11^ | 298 | 1.1×10^-11^ | NIST |  |
|  | O(3P) + NF_3_ | 1.40x10^-12^ exp(44/T) | 199 - 356 | 2.3×10^-11^ | JPL 19-5 |  |
|  | Products | 1.86x10^-11^ exp(44/T) |  |  | JPL 19-5 | Products unknown |

**Table S1.** Potential NF_3_ atmospheric destruction pathways.

| **Reactants** | **Products** | **Rate Law [cm3/molecule s]** | **Valid Temperature  Range [K]** | **Rate at 298K** | **Source** | **Note** |
| --- | --- | --- | --- | --- | --- | --- |
| SF_6_ + H | HF + SF_5_ | 3.32x10^-9^ exp(-15154.3/T) | 1460 - 1700 | N/A | NIST | High temp rxn |
| SF_6_ + CCl | Products | 2.41x10^-14^ | 298 | 2.41×10^-14^ | NIST | Products unknown |
| SF_6_ + CH | Products | <5.0x10^-17^ | 297 | N/A | NIST | Products unknown |
| SF_6_ | SF5 + F | 3.38x10^17^ (T/298)^-1.90 exp(-50754.9/T) | 1700 - 2000 | N/A | NIST | First-order reaction; Unit: [s-1] |
| CF_3_ + SF_6_ | CF_4_ + SF_5_ | 1.66x10^-13^ exp(-10100.5/T) | 303 - 638 | N/A | NIST |  |
| CH_3_ + SF_6_ | CH_3_F + SF_5_ | 3.32x10^-11^ exp(-7099.7/T) | 413 - 443 | N/A | NIST |  |
| O(1D) + SF_6_ | O(1D) + SF_6_ | 1.79x10^-14^ | 298 | 1.79×10^-14^ | NIST |  |
|  |  | N/A | | 1.80×10^-14^ | JPL 19-5 |  |

**Table S2.** Potential SF_6_ atmospheric destruction pathways.

| **Atom** | **Formula** | **Name** | **Boiling Point** | **Phase (at STP)** |
| --- | --- | --- | --- | --- |
| B | BF_3_ | Boron Trifluoride | −100.3 | gas |
| B | B_2_F_4_ | Diboron Tetrafluoride | −34 | gas |
| Si | SiF_4_ | Silicon Tetrafluoride | −90.3 | gas |
| Ge | GeF_4_ | Germanium Tetrafluoride | −36.5 | gas |
| N | NF_3_ | Nitrogen Trifluoride | −128.75 | gas |
| P | PF_3_ | Phosphorus Trifluoride | −101.8 | gas |
| P | P_2_F_4_ | Diphosphorus Tetrafluoride | −6.2 | gas |
| As | AsF_3_ | Arsenic Trifluoride | 57.8 | liquid |
| S | SF_6_ | Sulfur Hexafluoride | −50.8 | gas |
| Se | SeF_6_ | Selenium Hexafluoride | −34.5 | gas |

**Table S3.** Fully fluorinated molecules and their physical and chemical properties. STP is standard temperature and pressure.

**SI 4 Thermodynamics of Volcanic SF_6_ and NF_3_ Formation**

We have estimated the amount of SiF_4_, SF_6_ and NF_3_ that could be volcanically produced under a variety of atmospheric conditions. The conditions are those at which the gas erupts, and so is rapidly thermodynamically quenched into low temperature atmosphere, not necessarily conditions in the atmosphere. Below we show the analysis of all combinations of the following conditions:

- Temperature from 500K to 1500K in 100K steps
- 8 mineral redox buffers (QIF, IW, WM, IM, CoCoO, FMQ, NiNiO, MH)
- Abundance of water from 5% to 90% in 5% steps
- Pressure of 1, 10, 100, 1000, 10,000, 100,000 bar
- total gas sulfur content of 0.001, 0.01 or 0.1
- total gas HF content of 0.0001, 0.001, 0.01

The above conditions give a total of 85536 combinations. We assume that the 'activity' of SiO_2_ is 1, and that SiO_2_ is present as quartz: all other species are assumed to be gases. Abundances of H_2_, O_2_, H_2_S and SO_2_ are calculated from the oxygen fugacity (set by the mineral redox buffer) and the abundance of water and total sulfur.

Plotting the amount of SiF_4_ vs the amount of SF_6_ or NF_3_ shows that under almost all conditions the amount of SF_6_ and NF_3_ is less than SiF_4_ by many orders of magnitude (Figure S1).


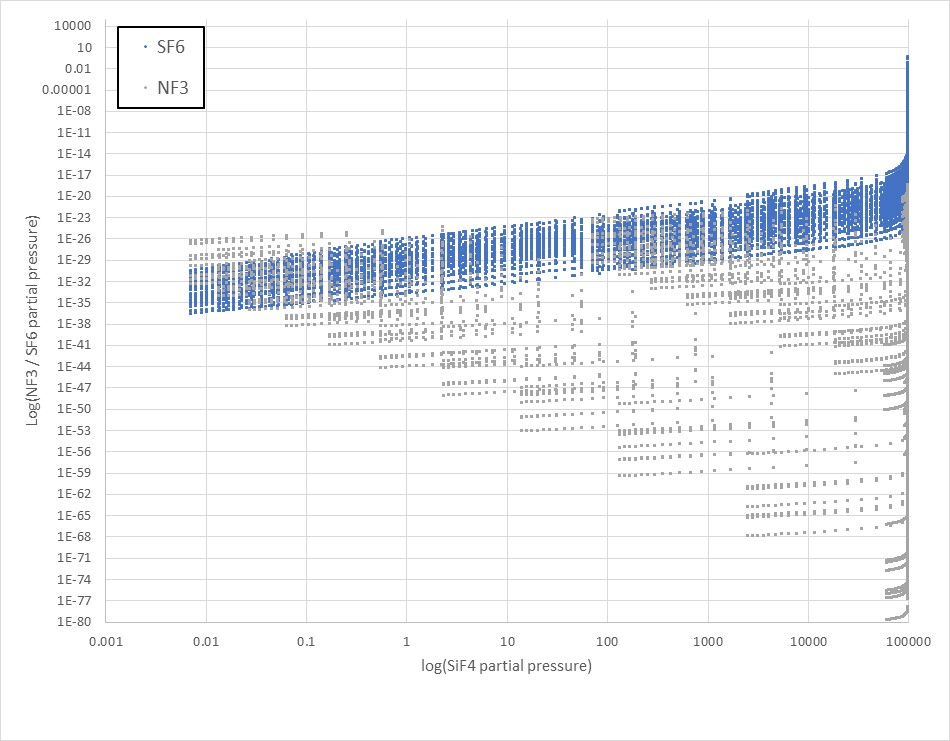


**Figure S1.** The amount of SiF_4_ compared to the amounts of NF_3_ and SF_6_ produced by volcanoes under 85,536 combinations of tested conditions. x axis: partial pressure of SiF_4_, y axis: partial pressure of SF_6_ (blue dots) and NF_3_ (grey dots). Under almost all conditions the amount of SF_6_ and NF_3_ is less than SiF_4_ by many orders of magnitude.

In a few sets of conditions shown on the Figure S1 the SF_6_ is a few % of SiF_4_ (top right area on the Figure S1). Those conditions are high pressure, low water, low temperature conditions where thermodynamics predicts that the atmosphere is composed almost entirely of SiF_4_, i.e. under those conditions the crustal rocks are being liquified and turned into SiF_4_. Such a scenario is physically and stoichiometrically implausible. (We assume an infinite reservoir of F in the rocks, which is implausible, although not impossible given the mass of the crust compared to the mass of the atmosphere.). We have therefore plot the abundance ratios of SF_6_/SiF_4_ and NF_3_/SiF_4_ (Figure S2). Plotting the ratios of SF_6_/SiF_4_ and NF_3_/SiF_4_ allows for the partial pressure of SiF_4_ to be arbitrary, therefore reflecting the ratios more accurately.


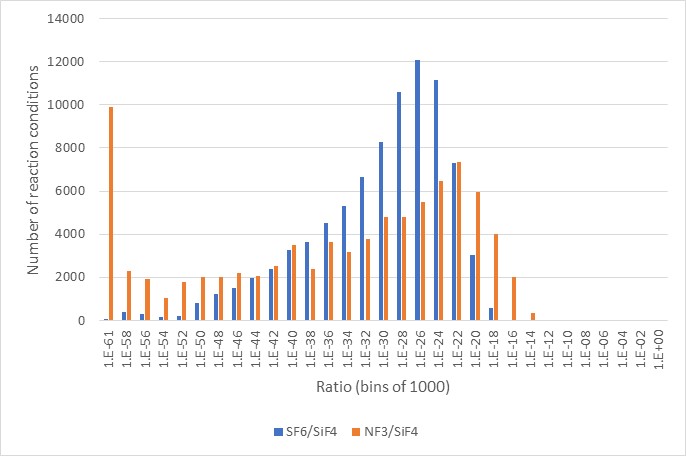

**Figure S2.** Abundance ratios of SF_6_/SiF_4_ and NF_3_/SiF_4_. y axis: number of reactions conditions. x axis: abundance ratios. No significant amounts of NF_3_ and SF_6_ are produced by volcanoes under 85,536 combinations of tested conditions.

**SI 5 Supplementary References**

1. Lin, H. W., Abad, G. G. & Loeb, A. Detecting industrial pollution in the atmospheres of earth-like exoplanets. *Astrophys. J. Lett.* **792**, L7 (2014).

2. Haqq-Misra, J. *et al.* Detectability of Chlorofluorocarbons in the Atmospheres of Habitable M-dwarf Planets. *Planet. Sci. J.* **3**, 60 (2022).

3. Haqq-Misra, J., Fauchez, T. J., Schwieterman, E. W. & Kopparapu, R. Disruption of a Planetary Nitrogen Cycle as Evidence of Extraterrestrial Agriculture. *Astrophys. J. Lett.* **929**, L28 (2022).

4. Kopparapu, R., Arney, G., Haqq-Misra, J., Lustig-Yaeger, J. & Villanueva, G. Nitrogen dioxide pollution as a signature of extraterrestrial technology. *Astrophys. J.* **908**, 164 (2021).

5. Sander, R. Compilation of Henry’s law constants (version 4.0) for water as solvent. *Atmos. Chem. Phys.* **15**, 4399–4981 (2015).

6. Golja, B., Barkanic, J. A. & Hoff, A. A review of nitrogen trifluoride for dry etching in microelectronics processing. *Microelectronics J.* **16**, 5–21 (1985).

7. Greenwood, N. N. & Earnshaw, A. *Chemistry of the Elements 2nd Edition*. (Butterworth-Heinemann, 1997).

8. Klapötke, T. M. Nitrogen–fluorine compounds. *J. Fluor. Chem.* **127**, 679–687 (2006).

9. Ishii, F. & Kita, Y. Chapter 19 - Applications of Fluorides to Semiconductor Industries. in (eds. Nakajima, T., Žemva, B. & Tressaud, A. B. T.-A. I. F.) 625–660 (Elsevier, 2000). doi:https://doi.org/10.1016/B978-044472002-3/50020-X.

10. Woytek, A. J., Lileck, J. T. & Barkanic, J. A. Nitrogen trifluoride―A new dry etchant gas. *Solid state Technol.* **27**, 172–175 (1984).

11. Greenberg, K. E. & Verdeyen, J. T. Kinetic processes of NF3 etchant gas discharges. *J. Appl. Phys.* **57**, 1596–1601 (1985).

12. Perrin, J., Méot, J., Siéfert, J.-M. & Schmitt, J. Mass spectrometric study of NF3 plasma etching of silicon. *Plasma Chem. plasma Process.* **10**, 571–587 (1990).

13. Elvers, B. *Ullmann’s encyclopedia of industrial chemistry*. vol. 17 (Verlag Chemie Hoboken, NJ, 1991).

14. Macintyre, J. E. *Dictionary of inorganic compounds*. (CRC Press, 1992).

15. Suslick, K. S. Kirk-Othmer encyclopedia of chemical technology. *J. Wiley Sons New York* **26**, 517–541 (1998).

16. Holleman, A. F., Wiberg, E. & Wiberg, N. *Inorganic chemistry*. (Academic press, 2001).

17. Atkins, P. & Overton, T. *Shriver and Atkins’ inorganic chemistry*. (Oxford University Press, USA, 2010).

18. Raj, G. *Advanced Inorganic Chemistry: Vollume II*. (Krishna Prakashan Media, 2010).

19. Lester, D. & Greenberg, L. A. The toxicity of sulfur hexafluoride. *Arch. Indust. Hyg. Occup. Med.* **2**, 348–349 (1950).

20. Dervos, C. T. & Vassiliou, P. Sulfur hexafluoride (SF6): global environmental effects and toxic byproduct formation. *J. Air Waste Manage. Assoc.* **50**, 137–141 (2000).

21. Ricketts, N. J. & Cashion, S. P. Hydrofluorocarbons as a replacement for sulphur hexafluoride in magnesium processing. *Magnes. Technol.* **2001**, 31–36 (2001).

22. Liptak, R. W., Devetter, B., Thomas, J. H., Kortshagen, U. & Campbell, S. A. SF6 plasma etching of silicon nanocrystals. *Nanotechnology* **20**, 35603 (2008).

23. Ottinger, D., Averyt, M. & Harris, D. US consumption and supplies of sulphur hexafluoride reported under the greenhouse gas reporting program. *J. Integr. Environ. Sci.* **12**, 5–16 (2015).

24. Dervos, C. T., Vassiliou, P. & Mergos, J. A. Thermal stability of SF6 associated with metallic conductors incorporated in gas insulated switchgear power substations. *J. Phys. D. Appl. Phys.* **40**, 6942 (2007).

25. Forster, P. *et al.* Changes in atmospheric constituents and in radiative forcing. Chapter 2. in *Climate change 2007. The physical science basis* (2007).

26. Prather, M. J. & Hsu, J. NF3, the greenhouse gas missing from Kyoto. *Geophys. Res. Lett.* **35**, (2008).

27. Zhang, X., Xiao, H., Tang, J., Cui, Z. & Zhang, Y. Recent advances in decomposition of the most potent greenhouse gas SF6. *Crit. Rev. Environ. Sci. Technol.* **47**, 1763–1782 (2017).

28. Myhre, G., Shindell, D. & Pongratz, J. Anthropogenic and natural radiative forcing. (2014).

29. Ray, E. A. *et al.* Quantification of the SF6 lifetime based on mesospheric loss measured in the stratospheric polar vortex. *J. Geophys. Res. Atmos.* **122**, 4626–4638 (2017).

30. Arnold, T. *et al.* Nitrogen trifluoride global emissions estimated from updated atmospheric measurements. *Proc. Natl. Acad. Sci.* **110**, 2029–2034 (2013).

31. Rigby, M. *et al.* Recent and future trends in synthetic greenhouse gas radiative forcing. *Geophys. Res. Lett.* **41**, 2623–2630 (2014).

32. Simmonds, P. G. *et al.* The increasing atmospheric burden of the greenhouse gas sulfur hexafluoride (SF 6). *Atmos. Chem. Phys.* **20**, 7271–7290 (2020).

33. Lide, D. R. CRC handbook of chemistry and physics, internet version 2005. (2005).
